# HER3- A key survival pathway and an emerging therapeutic target in metastatic colorectal cancer and pancreatic ductal adenocarcinoma

**DOI:** 10.18632/oncotarget.28421

**Published:** 2023-05-10

**Authors:** Omkar Desai, Rui Wang

**Affiliations:** ^1^Department of Surgery, Case Western Reserve University, Cleveland, OH 44106, USA; ^2^Case Comprehensive Cancer Center, Case Western Reserve University, Cleveland, OH 44106, USA; ^3^Department of Surgery, Division of Surgical Oncology, University Hospitals Cleveland Medical Center, Cleveland, OH 44106, USA

**Keywords:** HER3, colorectal, pancreatic cancer, metastasis, microenvironment

## Abstract

Colorectal cancer (CRC) and pancreatic ductal adenocarcinoma (PDAC) are highly metastatic cancers with poor survival rates. The tumor microenvironment has been shown to play a critical role in cancer progression and response to therapies. Endothelial cells (ECs) are a key component of the tumor microenvironment and promote cancer cell survival by secreting soluble factors that activate cancer-promoting signaling pathways. Studies from us and others identified HER3 as a key mediator of liver EC-induced chemoresistance and cancer cell growth in metastatic CRC and PDAC. In this article, we discuss that HER3-targeted therapies may be effective in treating patients with HER3-expressing CRC and PDAC, and highlight the importance of applying HER3 expression as a predictive biomarker for patient response to HER3-targeted therapies. We also discuss the challenges encountered in past clinical trials of HER3-targeted therapies, including the role of *NRG1* gene fusions, alternative HER3 activation mechanisms, and adaptive resistance mechanisms. Finally, we conclude by suggesting the future directions of HER3-targeted therapies, including novel approaches to overcome chemoresistance and promote cancer cell death.

Colorectal cancer (CRC) is the second leading cause of cancer-related deaths in the United States [1]. Approximately 20% of CRC cases are already metastatic at the time of diagnosis [2], while almost half of all patients with CRC eventually develop distant metastasis [3]. Pancreatic ductal adenocarcinoma (PDAC) is the third leading cause of cancer-related deaths in the United States and is predicted to become second within the next decade [4]. Nearly 50% of patients with PDAC have metastasis at the time of diagnosis and almost all patients with PDAC eventually develop distant metastasis [5–7]. Treatments are only marginally effective in patients with metastatic diseases, and the 5-year survival rates of metastatic CRC (mCRC) and metastatic PDAC (mPDAC) are 15.1% and 3.1%, respectively [2, 5]. Therefore, it is imperative to understand the regulation of cancer cell survival pathways in order to develop novel therapeutic strategies that can improve outcomes in patients with mCRC and mPDAC.

In the last decade, extensive research has been conducted to understand the effects of the microenvironment on cancer cells. Several preclinical studies have demonstrated that endothelial cells (ECs), a key component of the tumor microenvironment, promote cancer cell survival by secreting soluble factors in a paracrine fashion, which in turn activate cancer-promoting signaling pathways such as AKT, NF-κB and epithelial-mesenchymal transition pathway [8, 9]. The liver is the most common site of distant metastasis [10], with a metastatic prevalence of up to 80% in CRC and PDAC [11, 12]. It has a unique EC-rich microenvironment with up to 40% of the stroma being ECs [13–15]. Thus, the EC-rich microenvironment may affect metastatic cancer in the liver in a paracrine fashion. Following this logic, our laboratory recapitulated the liver EC microenvironment by isolating primary ECs from non-neoplastic liver tissues and established an *in vitro* model using liver EC conditioned medium (CM) to determine the effects of EC-secreted factors on CRC cells, with CM from CRC cells as controls. Using this model, we discovered that liver EC-secreted soluble factors increased cell proliferation and enhanced chemoresistance in CRC cells by activating survival pathways, such as AKT [16]. We further identified ERB-B2 receptor tyrosine kinase 3 (ErbB3, also known as HER3) as a key mediator of liver EC-induced chemoresistance and cell growth in CRC cells [16]. Consequently, administering a HER3 inhibitor, sapitinib (AZD8931), blocked the liver EC-induced CRC tumor growth in a subcutaneous (subQ) xenograft model [16]. Together these findings highlight the paracrine role of liver ECs in activating the HER3-AKT axis and promoting CRC survival. On a separate note, 40–50% of patients with mCRC harbor *KRAS* mutations, which lead to constitutive activation in downstream survival pathways [17, 18]. As a result, inhibitions of upstream effectors, such as HER3 and other ErbB receptors, have little effect on cancer cells with *KRAS* mutations. Indeed, therapies targeting epidermal growth factor receptor (EGFR), which is another member of the ErbB family, are rendered ineffective in patients with mCRC with *KRAS* mutations [18, 19]. Surprisingly, in our study we found that EC-induced activation of HER3 and downstream pathways in CRC is independent of *KRAS* mutation status [20]. As a result, HER3 inhibition led to significant anti-cancer effects in both *KRAS* wild-type and *KRAS*-mutant mCRC cells, and seribantumab, a humanized HER3 antibody, decreased tumor growth and sensitized tumors to fluorouracil chemotherapies in a liver injection orthotopic mCRC model [20]. These results suggest that HER3-targeted therapies may be useful in treating patients with *KRAS* wild-type or *KRAS*-mutant mCRC, either as monotherapy or in combination with standard-of-care chemotherapies.

Interestingly, the pro-survival paracrine role of liver ECs is also seen in other types of cancers that metastasize to the liver. We recently discovered that liver ECs secreted neuregulins (NRGs), which activated the HER3-AKT signaling axis in HER3-expressing mPDAC cells (HER3 +ve) [21]. Blocking the NRG-HER3 signaling axis with seribantumab effectively inhibited cell proliferation in HER3 +ve mPDAC [21]. Conversely, in mPDAC cells without HER3 expression (HER3 −ve), seribantumab was ineffective [21], thus suggesting an oncogenic role of liver ECs in PDAC that is independent of HER3. This body of work highlights that HER3-targeted therapies can be effective in treating patients with HER3 +ve mPDAC, and HER3 expression can be used as a predictive biomarker for patient response to HER3-targeted therapies.

In complement to our studies, prior preclinical studies have shown that HER3-targeted therapies with antibodies and inhibitors have been effective in blocking tumor growth in several types of cancers [22, 23], specifically breast cancer [24], head and neck squamous cell carcinoma (HNSCC) [25], PDAC [25], and non-small cell lung cancer (NSCLC) [26]. However, translating the preclinical findings to clinical studies has shown limited impact on patient outcomes. In a NSCLC clinical trial, the addition of HER3 antibody seribantumab to the standard of care EGFR inhibitor, erlotinib, showed no improvement in the progression-free survival compared to erlotinib alone [27]. Similar inconclusive results were seen in breast and ovarian cancer clinical trials (NCT01421472) (NCT01447706) [28]. However, a major limitation in these human studies was that appropriate predictive biomarkers such as HER3 expression were not used as an inclusion/exclusion criteria for patient enrollment. For example, only ~30% of primary PDAC and ~60% of mPDAC express HER3 [29, 30]. Based on our studies, only HER3 +ve cells and tumors are susceptible to HER3-targeted therapies [21]. Therefore failure of HER3-targeted therapies in previous clinical trials may have been due to a significant number of enrolled patients constituting HER3 −ve tumors. Outcomes of those trials remain controversial and warrants further investigation. On a positive note, in the aforementioned clinical trials, a small subset of patients with gene fusion mutations in neuregulin 1 (*NRG1*), the established HER3 ligand, responded exceptionally well to HER3-targeted therapies [27]. *NRG1* gene fusion mutations are caused by DNA rearrangements, resulting in the creation of a “chimera protein” by the fusion of NRG1 domains with partner proteins (such as ATP1B1, APP, C74, SDC4, and others) [31, 32]. As a result, *NRG1* fusion proteins are highly expressed on the cell membrane and induce HER3 activation. Following the encouraging data from patients with *NRG1* fusion mutations, a phase II clinical trial was initiated to assess the safety and efficacy of seribantumab in patients with advanced solid tumors that harbor *NRG1* gene fusion mutations (CRESTONE NCT04383210). Preliminary data from this clinical trial indicates that seribantumab has a favorable safety profile with a few dose-limiting toxicities and delivered durable response to seribantumab in patients with *NRG1* gene fusion, with an investigator-assessed objective response rate of 36% in NSCC and 33% in PDAC. These results suggest that *NRG1* fusions can be a predictive biomarker for patient response to HER3-targeted therapies. However, *NRG1* fusion mutations are extremely rare and occur in only ~0.2% of all solid tumors [32, 33]. Thus, identifying HER3 expression as another predictive biomarker for patient response to HER3-targeted therapies will have a significant and broad translational impact on treating patients with HER3 +ve solid tumors in future clinical trials.

An emerging perspective is that alternative HER3 activation mechanisms may be a reason for resistance to developed HER3 therapies. Recent unexpected findings suggest that other soluble factors secreted from the EC microenvironment activate HER3 in an NRG1-independent manner, potentially leading to a novel mechanism for HER3 activation that is independent from the canonical heterodimerization with other ErbB receptors, including EGFR and HER2 [16, 21, 34]. Our recent unpublished data suggests that EC-secreted leucine-rich α-2 glycoprotein 1 (LRG1) directly binds to and activates HER3 as a novel ligand [35]. It is possible that LRG1 binds to HER3 differently from NRGs. Hence, therapies such as seribantumab, which block NRG1-HER3 binding [36], may be ineffective. Further studies are warranted to investigate the HER3 signaling pathway and novel strategies aimed at blocking alternative HER3 activation mechanisms such as LRG1 need to be developed.

Furthermore, significant preclinical and clinical studies have determined that cancer cells may compensate for ErbB receptor inhibitions by overexpressing the non-targeted ErbB receptors as resistance mechanisms. This type of acquired resistance has been seen in lung tumors, where resistance to EGFR inhibitors (EGFRi) was associated with compensatory up-regulation of HER3, and combining EGFR and HER3 inhibitions led to improved anti-cancer effects compared to EGFRi alone [37]. Similar acquired resistance has been noted in breast cancer, where resistance to trastuzumab (HER2 antibody) was linked to upregulation of HER3 expression [38, 39]. As per this principle, it is possible that when treated with HER3 antibodies and inhibitors, cancer cells activate EGFR/HER2 or other alternative survival pathways to continue proliferation in a HER3-independent manner. Hence, the combination of blocking multiple ErbB family receptors may offer pharmacological opportunities to overcome resistance to a specific ErbB inhibition. However, distinct from the previously failed clinical trials, checking for HER3 expression/activation will be a key predictive biomarker to identify patients who may benefit from combination therapies in future clinical trials.

Novel HER3 antibody-drug conjugates (ADCs) have recently surfaced as an alternative strategy to improve the efficacy of HER3-targeted therapies. In these ADCs, antibody carriers provide targeted tumor cell delivery of cytotoxic drugs (payload). One such ADC is patritumab-deruxtecan, in which a HER3-specific antibody, patritumab, is attached to a topoisomerase I inhibitor payload [40]. Another HER3 antibody ADC uses a novel self-immolative T moiety for traceless conjugation and release of exatecan (topoisomerase I inhibitor) to overcome multidrug resistance in colon and lung cancer [41]. ADCs targeting HER3 may have an advantage over HER3 antibody monotherapy, since HER3-targeted ADCs only necessitate cancer cells to express HER3 for their cytotoxic effects to occur and are not solely dependent on blocking HER3. Developing additional ADCs using HER3 antibodies such as patritumab and seribantumab may be revolutionary in managing patients with HER3 +ve solid tumors.

In summary, we discovered that the surrounding liver EC microenvironment plays a key role in activating HER3 and promoting cell survival in mCRC and mPDAC, and potentially other types of cancer that metastasize to the liver. As HER3-targeted therapies have limited impact in patients with solid tumors in clinical trials to date, we speculate that lack of a predictive biomarker for patient response to therapies, alternative HER3 activation mechanisms, and adaptive resistance mechanisms such as upregulation of non-targeted ErbB receptors, may be some of the contributing factors. Therefore, identifying HER3 expression and/or activation as a predictive biomarker and elucidating alternative HER3 activation mechanisms will improve the efficacy of existing HER3-targeted therapies and help develop novel therapeutic strategies for blocking HER3-mediated survival pathways. Moreover, refining existing therapies by developing additional ADCs and combining HER3-targeted therapies with standard-of-care treatments may improve patient outcomes in future clinical trials. In conclusion, cancer treatment has witnessed a paradigm shift in recent years, and novel targeted therapeutic strategies continue to pave their way to disrupt the tumor microenvironment.
